# Proinflammatory Biomarkers and Clinical Factors Associated with Long-Term Mortality in People with HIV

**DOI:** 10.3390/v17020243

**Published:** 2025-02-11

**Authors:** Agnieszka Lembas, Andrzej Załęski, Tomasz Mikuła, Joanna Kozłowska, Alicja Wiercińska-Drapało

**Affiliations:** 1 Department of Infectious and Tropical Diseases and Hepatology, Medical University of Warsaw, 02-091 Warsaw, Poland; a.lembas@hotmail.com (A.L.);; 2 Hospital for Infectious Diseases in Warsaw, 01-201 Warsaw, Poland

**Keywords:** human immunodeficiency virus (HIV), mortality, proinflammatory biomarkers

## Abstract

People with HIV (PWH) receiving antiretroviral therapy (ART), despite a similar life expectancy, have a higher incidence of comorbidities than the general population. This study assessed the influence of proinflammatory biomarkers and clinical factors on mortality of PWH. We included PWH hospitalized from 2009 to 2014 who continued ART until 2023. The baseline lipid profile, CD4+ cell count, platelets, CRP, PCT, TNF-α, VCAM-1, and HCV and HBV coinfection were evaluated. Multivariable logistic regression was used to evaluate factors associated with mortality. Among 72 PWH, 19 were lost to a follow-up and 13 died before 2023. The mean follow-up was 12.07 years, while the mean time to death was 4.32 years. The main causes of death were cancer (*n* = 7) and drug-related death (*n* = 4). In the multivariate analysis, HCV coinfection, CRP ≥ 5 mg/L, PCT ≥ 0.05 ng/mL, and VCAM-1 ≥ 922 ng/mL were associated with higher odds of death. Although people who died had lower total cholesterol and triglyceride concentrations, these parameters were not associated with mortality. Determining HCV coinfections and CRP, PCT, and VCAM-1 levels may help identify PWH at increased risk of death for intensified monitoring. Care should also be taken of PWH with normal lipid parameters.

## 1. Introduction

Untreated human immunodeficiency virus (HIV) infection causes the progressive and continuous impairment of the immune system, resulting in the development of opportunistic infections and neoplasms, which are the clinical manifestations of acquired immunodeficiency syndrome (AIDS). Since the identification of HIV in 1983, approximately 40.1 million people have died of AIDS [1,2,3]. At the beginning of the global HIV pandemic, no antiretroviral therapy (ART) was available, which made AIDS-related diseases the main cause of death among people with HIV (PWH) [4]. Current studies suggest that PWH receiving effective antiretroviral therapy have similar life expectancies as the general population, albeit with a higher incidence of comorbidities [5].

The mechanisms by which the risk of comorbidities is increased in PWH are complex and multifactorial and include accelerated aging, lifestyle, ART toxicity, and other viral coinfections [6,7,8]. The chronic stimulation of inflammation by HIV itself plays an important role in the increased inflammatory status. Several molecules promote inflammation in HIV infection, including the C-reactive protein (CRP), whose concentrations are higher in PWH in comparison to the general population, and interleukin-6, whose high circulating levels are associated with HIV replication [9,10]. HIV also contributes to the production of interleukin-1β via the transformation of pro-interleukin-1β into bioactive interleukin-1β, which is associated with the progression to AIDS [11]. Replicating HIV induces a higher tumor necrosis factor alpha (TNF-α) serum concentration, and the overexpression of transforming growth factor beta (TGF-β) in PWH promotes viral replication and plays an important role in the progression of HIV infection [12,13]. Other proinflammatory factors in HIV are the adhesion molecules vascular cell adhesion molecule 1 (VCAM-1) and intercellular adhesion molecule 1 (ICAM-1), which mediate inflammation and promote leukocyte migration, whose expression is stimulated by the HIV-Tat-1 protein [14]. Other mechanisms currently being examined include CD4+ lymphocyte depletion, cellular reservoirs of HIV, increased intestinal permeability and gut microbiome impairment, and altered cholesterol and glucose metabolism. Another important proinflammatory mechanism involves cell activation biomarkers, including CD38 and HLA-DR. CD38 is a glycoprotein that plays an important role in regulating the differentiation and activation state of the cell. During early HIV infection, expression of the CD38 molecule is significantly increased. Higher expression levels of CD38 on CD4+ T lymphocytes are associated with more rapid development of AIDS [15]. The expression of HLA-DR on CD4+ T lymphocytes correlates with the level of total HIV DNA contributing to HIV persistence. HLA-DR+ cells are crucial in HIV infections because of their ability to release the virus when stimulated. The proportion of intact viral DNA sequences in the proliferative HLA-DR+ subset is high, which suggests they are critical in maintaining HIV infections [16].

PWH are also more prone to develop comorbidities because of unhealthy behaviors such as smoking, alcohol intake, and stimulant or other drug usage [17]. In people with HIV, people who inject drugs (PWID) may be especially vulnerable to concomitant diseases and have a higher risk of premature death for multiple reasons. For example, because of stigmatization, PWID may have limited access to HIV testing and antiretroviral therapy [18]. Moreover, PWID often have unstable lives and may be less likely to adhere to ART, which may result in higher proinflammatory molecule concentrations and a higher risk of comorbidities and mortality [19].

Effective ART leads to a decrease in the concentrations of the majority of proinflammatory molecules, although most remain higher than in the general population [20,21]. The prevalence of smoking, alcohol intake, and drug use in PWH remains higher than in the general population, increasing the risk of comorbidities, like neoplasms and cardiovascular disease (CVD), and premature death. This situation is particularly illustrated by the phenomenon that, in the general population, mortality rates from CVD are decreasing, whereas among PWH, they are increasing, mainly because of heart failure and stroke [22,23]. Despite more chronic conditions, PWH have a less pronounced increase in the risk of death than those with fewer conditions, compared with the general population [24]. This might be explained by the fact that PWH have multiple medical appointments due to HIV infection and, therefore, undergo regular and general health assessments and early treatment of comorbidities.

This study assessed the influence of proinflammatory biomarkers and clinical factors on long-term all-cause mortality in PWH treated with ART. An additional aim of the study was to evaluate factors impacting the mortality of PWH who inject drugs and HIV/HCV-coinfected individuals.

## 2. Materials and Methods

### 2.1. Patients

The study included the population of adult PWH hospitalized between 2009 and 2014. The inclusion criteria were HIV infection, regardless of the stage of the disease and time of treatment, continuing care in the HIV treatment center until 2023, and age 18 or older. The exclusion criteria were lipid-lowering therapy and signs of acute infection at the point of blood collection, including fever, cough, dyspnea, diarrhea, vomiting, dysuria, and skin infection. Patients receiving lipid-lowering agents were excluded in order to assess the differences in lipid parameters in between-group comparisons. Patients with infection were excluded to ensure that the elevation in proinflammatory biomarkers did not result from acute infection and may be assessed as a risk factor for long-term mortality.

### 2.2. Assessments

This was an observational, retrospective study. All patients underwent physical examination and laboratory testing. Each included patient was asked about the time of HIV infection diagnosis, the time of introduction of ART, concomitant disorders, and administered medications. Information about age, gender, education level, socio-economic status, and intravenous drug use was collected. Fasting blood samples were collected for total cholesterol, HDL cholesterol, LDL cholesterol, triglyceride level, CD4+ cell count, platelets, C-reactive protein, procalcitonin (PCT), TNF-α, and VCAM-1. The VCAM-1 and TNF-α serum levels were evaluated by enzyme-linked immunosorbent assay (Human sVCAM-1/CD106 Quantikine ELISA and Human TNF-alpha Quantikine ELISA, R&D Systems, respectively). All patients were assessed for hepatitis C virus (HCV) and hepatitis B virus (HBV) coinfection. HCV coinfection was confirmed by obtaining a positive HCV RNA, and HBV coinfection was confirmed by obtaining a positive HBs antigen or HBV DNA.

### 2.3. Long-Term Evaluation

All included patients were analyzed in 2023 in terms of death from any cause. Among patients who died, the time and cause of death were analyzed. The laboratory results and demographic data obtained in 2009–2014 were evaluated as long-term risk factors for death. The applied schemes of ART were not included in the analysis because of multiple switches in regimens during the observation period.

### 2.4. Statistical Analysis

The Shapiro–Wilk test was performed to verify the normality of the distributions in the analyzed variables. The two-sample Student *t*-test or Mann–Whitney U test was used to evaluate the difference in the mean values among quantitative variables, and χ^2^ or Fisher exact tests were performed for categorical variables. ROC curves and the Youden index were used to choose an appropriate cutoff point for VCAM-1 and TNF-α concentrations. Univariate and multivariable logistic regression analysis was used to evaluate the factors associated with odds of death during the observation period. Multivariable logistic regression was adjusted by age (<35 years old vs. ≥35 years old) and gender (male vs. female). The Kaplan–Meier estimator was used to estimate patients’ survival. The *p*-value was set at 0.05. All statistical analyses were performed using Python 3.7 software and Statistica 13.1 program (StatSoft Poland, Kraków, Poland).

### 2.5. Ethical Approval

The study was conducted in accordance with the Declaration of Helsinki and approved by the Ethics Committee of the Medical University of Warsaw, Poland (AKBE/128/2021).

## 3. Results

### 3.1. Patients

The study included 72 patients assessed in 2009–2014. Among them, 40 (55.56%) patients were alive in 2023, 13 (18.06%) patients had died, and 19 (26.39%) patients were lost at a follow-up. The reasons for the loss of a follow-up included moving to another treatment center (*n* = 10) and discontinuing care (*n* = 9). The mean follow-up was 12.07 years (min 9.22 years, max 13.78 years). In the analyzed cohort, 39 (54.17%) of the population were PWID. All PWID in the study had primary or secondary education and did not have regular employment. At baseline, one person was receiving anticoagulants because of deep vein thrombosis, two patients were receiving hypotensive drugs, and one patient was receiving oral antihyperglycemic treatment. Significant differences in analyzed parameters in the people who died and those who lived are presented in Table 1.

We observed significant differences in mean values of the duration of HIV infection, the presence of HCV coinfection, and serum concentrations of CRP, PCT, total cholesterol, triglycerides, and VCAM-1 among patients who lived and patients who died. All people diagnosed with HCV who stayed in care received HCV treatment with direct-acting antiviral agents with sustained virologic response at post-treatment week 24. Four patients died before the treatment was implemented.

### 3.2. Death

**Figure 1:**
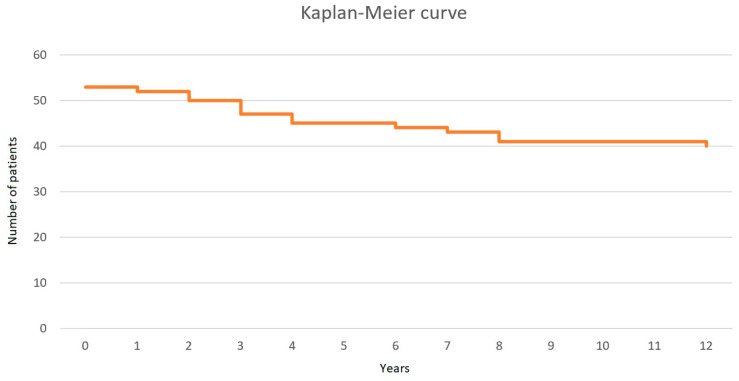
The Kaplan–Meier curve for study population survival.

Among patients who died, the mean time to death was 4.32 years (min. 0.34, max. 11.12 years). The causes of death were cancer (*n* = 7), drug-related deaths (*n* = 4), COVID-19 (*n* = 1), and septic shock (*n* = 1). Among patients who died of cancer, the causes of death were Hodgkin’s lymphoma (*n* = 2), esophageal cancer (*n* = 2), Kaposi sarcoma (*n* = 1), cervical cancer (*n* = 1), and bladder cancer (*n* = 1). There were no cancer diagnoses in patients who lived. In the group of people who died because of drug-related deaths, two individuals died of mental illness and two because of unintentional overdosing. The Kaplan–Meier curve is presented in Figure 1.

### 3.3. Factors Associated with the Risk of Death

**Figure 2:**
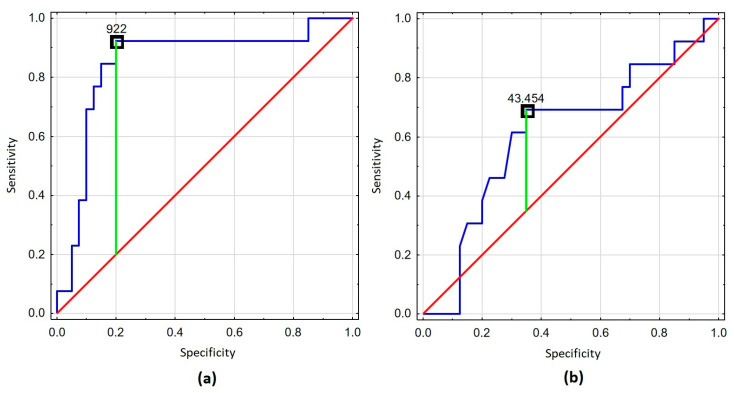
The ROC curves for VCAM-1 and TNF-α. (**a**) ROC curve for VCAM-1. (**b**) ROC curve for TNF-α.

We next analyzed whether clinical and immunological biomarkers were associated with the risk of death among people with HIV. We assessed the cutoff point of 922 ng/mL for VCAM-1 and 43.45 pg/mL for TNF-α using the Youden index. The remaining parameters were assessed according to normal limits. The ROC curves for VCAM-1 and TNF-α are presented in Figure 2.

The analyzed clinical factors associated with the risk of death are shown in Table 2.

The univariate analysis showed that age ≥35 years old, HCV coinfection, CRP ≥ 5 mg/L, PCT ≥ 0.05 ng/mL, VCAM-1 ≥ 922 ng/mL, and HDL cholesterol ≤ 1 mmol/L were associated with higher odds of death. In the multivariable analysis, only HCV coinfection, CRP ≥ 5 mg/L, PCT ≥ 0.05 ng/mL, and VCAM-1 ≥ 922 ng/mL were significant risk factors for death.

### 3.4. Factors Associated with Mortality of PWID

The majority (54.17%) of the study population declared intravenous drug use. In this population, 11 patients died. The causes of death were cancer (*n* = 5), drug-related deaths (*n* = 4), COVID-19 (*n* = 1), and septic shock (*n* = 1). We analyzed factors associated with the risk of death among PWH who inject drugs. The results are presented in Table 3.

Among PWH who inject drugs, only HCV coinfection and CRP ≥ 5 mg/L were significant risk factors for death.

### 3.5. Factors Associated with Cancer Incidence

Cancer was the primary cause of death in the study population. There were no cases of cancer in patients who lived. We evaluated factors associated with the risk of death due to cancer among PWH. The results are presented in Table 4.

In our analysis, PCT ≥ 0.05 ng/mL and VCAM-1 ≥ 922 ng/mL were independent risk factors for cancer incidence among PWH.

### 3.6. Differences in Analyzed Parameters in PWH with HCV Coinfection

Since HCV coinfection was a significant risk factor for death in PWH and also in PWH who inject drugs, we evaluated the differences in mean analyzed parameters between HIV/HCV-coinfected individuals who died and those who lived. In the analyzed subpopulation, there were eight people who died and ten people who lived. The results are presented in Table 5.

Individuals with HIV/HCV coinfection who died had significantly higher concentrations of VCAM-1 and significantly lower concentrations of HDL cholesterol.

## 4. Discussion

### 4.1. Cause of Death Among PWH

The study was a long-term observation of all-cause mortality of PWH receiving ART. From the initial study population of 72 patients, we observed 74%. Among them, 25% died during the observation period. The main cause of death was cancer and drug-related deaths. According to current research, the most prevalent causes of death of PWH include AIDS, non-AIDS malignancy, and cardiovascular reasons. Although AIDS is still the main cause of death in untreated PWH, the rates of death due to AIDS are declining [25]. In our cohort, there were no deaths due to AIDS; however, our study included only patients who were receiving ART and regularly attended follow-up. The majority of deaths were due to cancer, which corresponds with current research [25]. In our analysis, cancer incidence in PWH was related to increased levels of PCT and VCAM-1. Our findings correspond with current research, and procalcitonin is being evaluated as a prognostic marker of cancer patients [26]. Increased PCT concentrations may be an indication of non-small cell lung cancer and may be useful in the follow-up of medullary thyroid cancer [27,28]. VCAM-1 may be linked to the development of cancer [29], and elevated concentrations of VCAM-1 have been found in the peripheral blood of patients with cancer [30].

The second most prevalent cause of death in our population was drug-related death; 30% of deaths were related to intravenous drug use (mental illness in people who inject drugs (15%) and drug abuse (15%)). One study showed that PWH who inject drugs have higher mortality rates than PWH who do not inject drugs, and in this population, drug-related deaths accounted for 45.5% of all deaths [31]. In our cohort, PWID accounted for over 50% of all analyzed patients. In this population, HCV coinfection and CRP ≥ 5 mg/L were independent risk factors for mortality. HCV coinfection is prevalent in PWID since it is estimated that 52.3% of all PWID are HCV-antibody positive [32]. Its incidence enhances the mortality of PWID since HCV infection is a known risk factor for mortality and cancer, especially in HIV/HCV-coinfected individuals [33]. CRP levels of PWID with no acute infection have not been extensively studied; however, it is possible that PWID experience increased levels of proinflammatory biomarkers resulting from the detectable HIV viral load that is prevalent in this population [34].

### 4.2. Possible Mechanisms of Premature Death Among PWH

#### 4.2.1. Proinflammatory Biomarkers

One possible explanation of mortality in PWH is proinflammatory mechanisms. It was observed that PWH have higher levels of proinflammatory biomarkers than the general population. Studies show elevated CRP, D-Dimer, interleukins 1β, 6, 11, and 27, TNF-α, TGF-β, cell adhesion molecules, and others in PWH compared to healthy controls [35,36,37,38]. Even when receiving ART and with a suppressed HIV viral load, the levels of these biomarkers are still elevated compared to healthy controls [35,36,37].

A chronic increase in CRP levels, one of the most prevalent markers of inflammation, is also a well-known risk factor for CVD and a predictor of all-cause mortality [38,39]. There are reports suggesting that higher levels of CRP may be related not only to CVD and all-cause mortality but also to higher odds of death due to cancer, which is a very common cause of death in PWH [40]. In PWH, increased levels of CRP are also associated with a higher HIV viral load, microbial translocation, immune activation, and mortality [9]. Based on the former research, we evaluated whether the elevation of CRP concentration may also be a long-term mortality predictor in PWH. We found that CRP ≥ 5 mg/L in PWH with no signs of acute infection was associated with higher odds of death. Our findings correspond with research suggesting that higher baseline CRP in PWH is associated with higher all-cause mortality [41]. Studies also indicate that even people with CD4+ cell count > 500 cells/µL experience intensified proinflammatory processes, resulting in an increase in CRP concentrations and mortality. People with HIV with CRP > 3 mg/L had 2.7-fold higher odds of death during the 5-year observation period than those with CRP < 1 mg/L [42]. In our analyses, CRP correlated with higher odds of death in the entire PWH cohort, as well as in the subgroup of PWH who inject drugs. It was observed that PWID experience higher levels of CRP, which also might contribute to increased vulnerability to death [43].

Procalcitonin, together with CRP, is a marker for systemic inflammation and an important diagnostic marker of sepsis that is used for the diagnosis and prediction of the severity of infection [44]. PCT may also be produced because of noninfectious causes, such as surgery, shock, burn injury, trauma, chronic kidney disease, and liver cirrhosis [44,45]. There are reports that suggest that an increased PCT level is a predictive marker of mortality in trauma and also an independent risk factor associated with poor prognosis in cancer patients [26,46]. There are few studies evaluating the predictive value of PCT in HIV infection. The studies assessing the impact of PCT concentrations on long-term mortality in patients with no acute infection are also limited. In a study by Zhang et al., elevated PCT levels were identified as an independent risk factor for mortality of HIV/*Talaromyces marneffei* coinfection [47]. Osawa et al. showed that increased PCT levels may predict mortality in tuberculosis patients with no HIV infection [48]. Our research corresponds with these findings since we observed significantly higher odds of death among patients with PCT serum concentrations of ≥0.5 ng/dL. To our knowledge, our study is the first to evaluate PCT concentrations as a long-term mortality risk in PWH with no acute infection.

VCAM-1 is a cell adhesion molecule [49]. Chronic overexpression of VCAM-1, which was observed in PWH, promotes proatherogenic mechanisms and increases cardiovascular risk [50]. We observed that VCAM-1 ≥ 922 ng/mL was associated with higher mortality rates. Our findings correspond with other research, which suggests that VCAM-1 may be a biomarker for predicting the risk of HIV-1 disease progression, morbidity, and mortality [51]. In a study by Affi et al., VCAM-1 baseline concentrations ≥ 1458 ng/mL were associated with higher mortality rates of PWH during 30 months of observation [52]. Interestingly, in PWH, VCAM-1 levels before ART implementation were significantly associated with mortality, independently of whether ART was started immediately or deferred [35]. VCAM-1 is also currently being evaluated as a biomarker of mortality in other populations. For example, elevated VCAM-1 increases the risks of sepsis, septic shock, multiple-organ dysfunction syndrome, and death [53].

#### 4.2.2. HCV Coinfection

Our analysis showed that concomitant HCV coinfection is associated with higher mortality rates in comparison to HIV mono-infection. The elevation in levels of plasma inflammation and microbial translocation biomarkers, especially soluble CD14 and interleukin-6 in people with HIV/HCV coinfection compared to PWH, have also been previously observed [54]. HCV-related liver diseases remain a significant cause of morbidity, in-hospital treatment, and mortality of PWH, as HIV/HCV-coinfected individuals experience an accelerated progression of chronic liver disease and higher mortality [55,56]. Moreover, HIV/HCV-coinfected individuals have a higher risk of all-cause and non-liver-related deaths and non-liver-related cancers than HCV mono-infected patients [33]. Morbidity and mortality in this population remain high due to metabolic disorders associated with the occurrence of cardiovascular events [57]. In our analysis, people with HIV/HCV coinfection who died had significantly higher concentrations of VCAM-1 than those who lived. The elevation of VCAM-1 concentrations in HCV coinfection is common and seems to decrease with HCV eradication [58]. Limited studies also suggest that VCAM-1 may predict liver-related events and death in patients with HIV/HCV coinfection [59].

#### 4.2.3. CVD and Lipid Profile

Since CVD is one of the main causes of death in PWH, we evaluated the lipid profiles among the study group and observed that in people who died, the concentration of total cholesterol and triglycerides was significantly lower compared to those who lived. However, in multivariable analysis, lipid parameters were not associated with the odds of death. It has been well documented that a significant portion of PWH have impaired lipid profiles that get worse during ART [60]. Hypothetically, our results might be explained by the fact that 62% of people who died were coinfected with HCV, and chronic HCV infection is associated with low levels of lipid parameters, especially VLDL and LDL. Patients with HCV infection, despite a seemingly beneficial lipid profile, still have accelerated development of atherosclerosis, leading to an increased cardiovascular risk [61].

#### 4.2.4. Other Evaluated Risk Factors for Premature Death in PWH

In our study, age, gender, HBV coinfection, CD4+ cell count, platelet count, and TNF-α were not correlated with the odds of death. According to current research, HIV/HBV coinfection is associated with increased mortality [62]. Our study group may have included too few patients with HBV coinfection to observe this relationship. Our analysis included only incidental CD4+ cell count measurements, and studies show that the CD4+ lymphocyte nadir is a more important parameter to predict mortality following noncommunicable diseases in PWH [63]. To our knowledge, even though platelets are involved in inflammatory reactions, there are no direct reports of their impact on mortality [64]. However, there are suggestions that platelets containing HIV may contribute to a worse immunological response to ART [65]. Moreover, platelet activation, which is often increased in PWH, may stimulate neutrophil–platelet–endothelium interactions and, therefore, increase the risk of CVD. This may indirectly be associated with morbidity and mortality of PWH [66]. Interestingly, in our cohort, the levels of TNF-α were lower in people who died compared to those who lived. Some studies show that the levels of TNF-α in PWH remain high even with long-term ART, but its impact on mortality is currently unknown [67]. Other research suggests that HIV infection does not have any effect on TNF-α levels, regardless of treatment status [68].

In the future, we hope to evaluate additional biomarkers, such as D-dimer, interleukins (including 1β, 6, 11, and 27), and TGF-β as PWH mortality risk factors. D-dimer was the most predictive biomarker of overall mortality in PWH out of a panel of inflammatory and coagulation biomarkers [69]. Moreover, among HIV/HCV-coinfected patients initiating ART, D-dimer was also associated with an increased risk of death [70]. Elevated concentrations of interleukins, especially interleukin-6, have been linked with higher mortality rates in men with HIV with undetectable HIV viral loads [71]. TGF-β expression is an important cancer prognostic factor, and HIV-1 Tat protein triggers TGF-β production; therefore, investigating its association with PWH mortality is a promising approach [72,73].

### 4.3. Significance and Limitations of the Study

Our study is significant because of its long follow-up (12 years) and the analysis of multiple biomarkers related to the mortality of PWH. Among analyzed biomarkers, we evaluated PCT in a long-term assessment of PWH, excluding acute patients, which, to our knowledge, has not previously been evaluated as a factor associated with long-term mortality in PWH. However, our study has several limitations. Firstly, the study cohort was relatively small in reliably evaluating the association between the analyzed biomarkers and PWH mortality, which was exacerbated by the fact that we followed up with only 74% of the initial study population. The loss of 26% of patients to follow-up can lead to selection bias, and the variety of analyzed biomarkers in a small study population may pose a risk of type II errors. Secondly, the measurement of analyzed biomarkers was performed only once. Although we included only patients with no signs of acute infection, using single measurements may impact the results. Another limitation is the lack of analysis of ART regimens as a potential risk factor for death, which was impossible to perform because of multiple switches in a limited group of patients. Nevertheless, we believe our observations are an overture to further research using larger populations.

## 5. Conclusions

Long-term mortality in PWH may be connected to high CRP, PCT, and VCAM-1 concentrations, as well as HCV coinfection. Determining CRP, PCT, and VCAM-1 levels may help identify PWH at increased long-term risk of death for intensified monitoring. PWH should be regularly assessed for HCV coinfection. Care should also be taken for PWH with normal lipid parameters since a beneficial lipid profile is not a sufficient factor to reduce the odds of death.

## Figures and Tables

**Table 1:** Baseline data of all analyzed patients.

Variable	All Patients (*n* = 72)	Patients Who Lived (*n* = 40)	Patients Who Died (*n* = 13)	*p*
Age (years) (mean (SD))	38.68 (11.04)	37.73 (11.42)	40.85 (11.52)	0.153
Male gender (n (%))	56 (77.78)	31 (77.50)	11 (84.62)	0.711
Female gender (n (%))	16 (22.22)	9 (22.50)	2 (15.38)	0.711
Intravenous drug use (n (%))	39 (54.17)	18 (45.00)	11 (84.62)	0.013
HCV coinfection (n (%))	26 (36.11)	10 (25.00)	8 (61.54)	0.022
HBV coinfection (n (%))	13 (18.06)	6 (15.00)	4 (30.77)	0.237
Duration of HIV infection (years) (mean (SD))	4.48 (5.21)	2.80 (3.11)	7.54 (6.80)	0.001
Duration of HIV treatment (weeks) (mean (SD))	93.50 (142.28)	76.28 (126.44)	116.61 (181.23)	0.355
PLT (G/L) (mean (SD))	217.82 (99.59)	225.88 (111.34)	182.46 (90.98)	0.209
CRP (mg/L) (mean (SD))	22.10 (44.61)	10.75 (15.15)	39.17 (50.42)	0.003
PCT (ng/mL) (mean (SD))	0.72 (3.98)	0.05 (0.01)	0.36 (0.60)	0.002
VCAM-1 (ng/mL) (mean (SD))	912.00 (68.52)	729.10 (556.28)	1574.54 (770.91)	<0.001
TNF-α (pg/mL) (mean (SD))	64.55 (64.99)	67.96 (79.82)	48.71 (24.96)	0.113
CD4+ cell count (cells/μL) (mean (SD))	294.47 (210.60)	280.95 (204.23)	191.31 (111.84)	0.085
Total cholesterol (mmol/L) (mean (SD))	4.18 (1.20)	4.27 (0.95)	3.41 (1.28)	0.011
LDL cholesterol (mmol/L) (mean (SD))	2.43 (1.00)	1.58 (0.83)	1.69 (0.76)	0.316
HDL cholesterol (mmol/L) (mean (SD))	1.15 (0.41)	2.30 (0.82)	1.95 (0.93)	0.120
Triglycerides (mmol/L) (mean (SD))	1.70 (0.86)	1.18 (0.35)	0.82 (0.28)	0.002

**Table 2:** Factors associated with the risk of death in people with HIV by univariate and multivariable logistic regression analysis. The analysis compared patients who died with those who lived who stayed in care for the whole observation period.

Parameter	OR (95% CI)	*p*	aOR * (95% CI)	*p*
Age				
<35 years (*n* = 23)	Ref.			
≥35 years (*n* = 30)	6.08 (1.19–31.01)	0.030	4.01 (0.75–21.49)	0.104
Gender				
Male (*n* = 42)	Ref.			
Female (*n* = 11)	2.86 (0.32–25.80)	0.350	2.84 (0.31–26.76)	0.362
Intravenous drug use				
No (*n* = 24)	Ref.			
Yes (*n* = 29)	6.72 (1.32–34.32)	0.009	6.59 (1.22–35.66)	0.029
HCV infection				
No (*n* = 35)	Ref.			
Yes (*n* = 18)	4.80 (1.27–18.09)	0.021	5.64 (1.17–27.22)	0.031
HBV infection				
No (*n* = 43)	Ref.			
Yes (*n* = 10)	2.52 (0.58–10.88)	0.216	2.46 (0.51–11.85)	0.262
CD4+ cell count				
≥200 cells/μL (*n* = 35)	Ref.			
<200 cells/μL (*n* = 17)	1.46 (0.40–5.38)	0.571	1.70 (0.37–7.91)	0.498
PLT				
≥140 G/L (*n* = 40)	Ref.			
<140 G/L (*n* = 13)	1.53 (0.38–6.16)	0.549	2.63 (0.52–13.29)	0.242
CRP				
<5 mg/L (*n* = 28)	Ref.			
≥5 mg/L (*n* = 25)	5.56 (1.32–23.46)	0.020	20.65 (1.88–227.24)	0.013
PCT				
<0.05 ng/mL (*n* = 44)	Ref.			
≥0.05 ng/mL (*n* = 9)	22.17 (3.69–133.02)	<0.001	16.60 (2.43–113.20)	0.004
VCAM-1				
<922 ng/mL (*n* = 33)	Ref.			
≥922 ng/mL (*n* = 20)	48.00 (5.41–425.56)	<0.001	33.49 (3.54–316.98)	0.002
TNF-α				
<43.45 pg/mL (*n* = 22)	Ref.			
≥43.45 pg/mL (*n* = 31)	0.34 (0.09–1.23)	0.099	0.30 (0.07–1.35)	0.116
Total cholesterol				
<4.91 mmol/L (*n* = 44)	Ref.			
≥4.91 mmol/L (*n* = 9)	0.33 (0.04–2.96)	0.324	0.24 (0.00–5.64)	0.997
HDL cholesterol				
>1 mmol/L (*n* = 34)	Ref.			
≤1 mmol/L (*n* = 19)	4.22 (1.13–15.72)	0.032	2.67 (0.62–11.56)	0.188
LDL cholesterol				
<2.6 mmol/L (*n* = 40)	Ref.			
≥2.6 mmol/L (*n* = 13)	0.19 (0.02–1.67)	0.135	0.17 (0.00–3.78)	0.998
Triglyceride				
<1.7 mmol/L (*n* = 38)	Ref.			
≥1.7 mmol/L (*n* = 15)	1.88 (0.50–7.07)	0.353	1.60 (0.35–7.28)	0.543

* aOR—adjusted odds ratio. The odds ratio was adjusted in the multivariate logistic model by age (<35 years old vs. ≥35 years old) and gender (male vs. female).

**Table 3:** Factors associated with the risk of death in PWH who inject drugs by univariate and multivariable logistic regression analysis. The analysis compared patients who died and those who lived who stayed in care for the whole observation period.

Parameter	OR (95% CI)	*p*	aOR * (95% CI)	*p*
Age				
<35 years (*n* = 11)	Ref.			
≥35 years (*n* = 18)	4.50 (0.75–26.93)	0.100	4.93 (0.73–33.21)	0.764
Gender				
Male (*n* = 23)	Ref.			
Female (*n* = 7)	1.50 (0.43–5.21)	0.523	1.72 (0.08–6.18)	0.362
HCV infection				
No (*n* = 15)	Ref.			
Yes (*n* = 14)	5.33 (1.03–27.76)	0.047	6.18 (1.03–36.89)	0.046
HBV infection				
No (*n* = 22)	Ref.			
Yes (*n* = 7)	1.31 (0.23–7.41)	0.758	1.01 (0.14–7.46)	0.996
CD4+ cell count				
≥200 cells/μL (*n* = 22)	Ref.			
<200 cells/μL (*n* = 7)	2.86 (0.50–16.36)	0.238	2.84 (0.37–21.57)	0.314
PLT				
≥140 G/L (*n* = 24)	Ref.			
<140 G/L (*n* = 5)	3.00 (0.41–21.74)	0.277	2.44 (0.27–21.97)	0.426
CRP				
<5 mg/L (*n* = 13)	Ref.			
≥5 mg/L (*n* = 16)	7.07 (1.17–42.85)	0.033	9.42 (1.30–68.01)	0.026
PCT				
<0.05 ng/mL (*n* = 6)	Ref.			
≥0.05 ng/mL (*n* = 23)	7.06 (0.00–250.09)	0.997	25.09 (0.00–286.89)	0.997
VCAM-1				
<922 ng/mL (*n* = 14)	Ref.			
≥922 ng/mL (*n* = 15)	4.50 (0.75–26.93)	0.099	3.28 (0.36–35.78)	0.994
TNF-α				
<43.45 pg/mL (*n* = 19)	Ref.			
≥43.45 pg/mL (*n* = 10)	0.47 (0.14–1.56)	0.218	0.29 (0.05–1.74)	0.117
Total cholesterol				
<4.91 mmol/L (*n* = 25)	Ref.			
≥4.91 mmol/L (*n* = 4)	0.50 (0.05–5.51)	0.571	0.26 (0.02–3.17)	0.288
HDL cholesterol				
>1 mmol/L (*n* = 17)	Ref.			
≤1 mmol/L (*n* = 12)	4.55 (0.92–22.63)	0.064	3.28 (0.60–17.98)	0.172
LDL cholesterol				
<2.6 mmol/L (*n* = 21)	Ref.			
≥2.6 mmol/L (*n* = 8)	0.16 (0.02–1.51)	0.109	0.06 (0.01–0.69)	0.052
Triglyceride				
<1.7 mmol/L (*n* = 21)	Ref.			
≥1.7 mmol/L (*n* = 8)	2.00 (0.38–10.48)	0.412	1.89 (0.30–11.94)	0.497

* aOR—adjusted odds ratio. The odds ratio was adjusted in the multivariate logistic model by age (<35 years old vs. ≥35 years old) and gender (male vs. female).

**Table 4:** Factors associated with the risk of cancer incidence in PWH—univariate and multivariable logistic regression analysis. The analysis compared patients who died and those who lived who stayed in care for the whole observation period.

Parameter	OR (95% CI)	*p*	aOR * (95% CI)	*p*
Age				
<35 years (*n* = 22)	Ref.			
≥35 years (*n* = 25)	6.63 (0.73–60.22)	0.093	7.86 (0.80–77.16)	0.077
Gender				
Male (*n* = 36)	Ref.			
Female (*n* = 11)	0.73 (0.12–4.39)	0.726	0.45 (0.06–3.29)	0.434
Intravenous drug use				
No (*n* = 24)	Ref.			
Yes (*n* = 23)	3.06 (0.53–17.66)	0.212	3.00 (0.48–18.73)	0.239
HCV infection				
No (*n* = 33)	Ref.			
Yes (*n* = 14)	4.00 (0.76–21.02)	0.102	6.89 (1.00–46.69)	0.051
HBV infection				
No (*n* = 39)	Ref.			
Yes (*n* = 8)	2.27 (0.35–14.49)	0.387	1.37 (0.18–10.27)	0.759
CD4+ cell count				
≥200 cells/μL (*n* = 31)	Ref.			
<200 cells/μL (*n* = 16)	4.67 (0.75–29.01)	0.098	4.39 (0.65–29.53)	0.128
PLT				
≥140 G/L (*n* = 34)	Ref.			
<140 G/L (*n* = 13)	4.59 (0.86–24.42)	0.074	5.15 (0.76–34.84)	0.093
CRP				
<5 mg/L (*n* = 27)	Ref.			
≥5 mg/L (*n* = 20)	4.17 (0.72–24.23)	0.112	4.41 (0.70–27.84)	0.115
PCT				
<0.05 ng/mL (*n* = 41)	Ref.			
≥0.05 ng/mL (*n* = 6)	25.33 (3.21–199.69)	0.002	18.41 (1.84–183.98)	0.013
VCAM-1				
<922 ng/mL (*n* = 33)	Ref.			
≥922 ng/mL (*n* = 14)	24.00 (2.52–228.70)	0.006	19.09 (1.92–189.82)	0.012
TNF-α				
<43.45 pg/mL (*n* = 20)	Ref.			
≥43.45 pg/mL (*n* = 27)	0.09 (0.01–0.82)	0.033	0.11 (0.01–1.08)	0.058
Total cholesterol				
<4.91 mmol/L (*n* = 38)	Ref.			
≥4.91 mmol/L (*n* = 9)	0.67 (0.07–6.35)	0.724	9.18 (0.90–93.99)	0.465
HDL cholesterol				
>1 mmol/L (*n* = 32)	Ref.			
≤1 mmol/L (*n* = 15)	3.52 (0.68–18.30)	0.135	2.97 (0.51–17.17)	0.225
LDL cholesterol				
<2.6 mmol/L (*n* = 34)	Ref.			
≥2.6 mmol/L (*n* = 13)	0.39 (0.04–3.59)	0.405	0.22 (0.02–2.26)	0.205
Triglyceride				
<1.7 mmol/L (*n* = 34)	Ref.			
≥1.7 mmol/L (*n* = 13)	2.25 (0.43–11.83)	0.338	2.32 (0.35–15.30)	0.380

* aOR—adjusted odds ratio. The odds ratio was adjusted in the multivariate logistic model by age (<35 years old vs. ≥35 years old) and gender (male vs. female).

**Table 5:** Differences in analyzed parameters in HIV/HCV-coinfected individuals depending on mortality. The analysis compared patients who died and those who lived who stayed in care for the whole observation period.

Variable	All HIV/HCV-Coinfected Patients (*n* = 18)	Patients Who Lived (*n* = 10)	Patients Who Died (*n* = 8)	*p*
Age (years) (mean (SD))	35.94 (6.66)	33.80 (6.81)	38.63 (5.78)	0.130
Male gender (n (%))	14 (77.78)	8 (80.00)	6 (75.00)	0.800
Female gender (n (%))	4 (22.22)	2 (20.00)	2 (25.00)	0.800
Intravenous drug use (n (%))	14 (77.78)	6 (60.00)	8 (100.00)	0.196
HBV coinfection (n (%))	3 (16.67)	1 (10.00)	2 (25.00)	0.466
Duration of HIV infection (years) (mean (SD))	5.42 (6.53)	2.65 (3.73)	8.88 (7.81)	0.322
Duration of HIV treatment (weeks) (mean (SD))	86.39 (168.59)	82.50 (176.06)	91.25 (170.67)	0.427
PLT (G/L) (mean (SD))	215.17 (128.37)	236.20 (156.64)	188.88 (83.96)	0.454
CRP (mg/L) (mean (SD))	18.83 (23.32)	14.30 (23.36)	24.50 (24.66)	0.252
PCT (ng/mL) (mean (SD))	0.11 (0.20)	0.05 (0.00)	0.17 (0.30)	0.056
VCAM-1 (ng/mL) (mean (SD))	1234.67 (925.33)	797.60 (786.00)	1781.00 (819.19)	0.002
TNF-α (pg/mL) (mean (SD))	54.87 (23.46)	56.49 (21.11)	52.84 (27.49)	0.374
CD4+ cell count (cells/μL) (mean (SD))	253.89 (203.67)	313.00 (258.00)	180.00 (62.66)	0.504
Total cholesterol (mmol/L) (mean (SD))	3.88 (1.02)	4.07 (0.82)	3.65 (1.24)	0.395
LDL cholesterol (mmol/L) (mean (SD))	2.14 (0.74)	2.06 (0.59)	2.23 (0.93)	0.924
HDL cholesterol (mmol/L) (mean (SD))	1.04 (0.36)	1.25 (0.28)	0.78 (0.27)	0.002
Triglycerides (mmol/L) (mean (SD))	1.72 (0.89)	1.65 (0.91)	1.80 (0.91)	0.964

## Data Availability

Data are contained within the article.

## Abbreviations

The following abbreviations are used in this manuscript:

PWH	People with HIV
ART	Antiretroviral therapy
HIV	Human immunodeficiency virus
AIDS	Acquired immunodeficiency syndrome
CVD	Cardiovascular disease
CRP	C-reactive protein
PCT	Procalcitonin
TNF-α	Tumor necrosis factor alpha
VCAM-1	Vascular cell adhesion molecule 1
ICAM-1	Intercellular adhesion molecule 1
HCV	Hepatitis C virus
HBV	Hepatitis B virus
PLT	Platelets
PWID	People who inject drugs
TGF-β	Transforming growth factor beta
